# Fear in urban landscapes: conspecific flock size drives escape decisions in tropical birds

**DOI:** 10.1098/rsos.221344

**Published:** 2022-11-30

**Authors:** Melissa Ardila-Villamizar, Gustavo Alarcón-Nieto, Adriana A. Maldonado-Chaparro

**Affiliations:** ^1^ Department of Biology, Faculty of Natural Sciences, Universidad del Rosario, Bogota D.C., Colombia; ^2^ Department of Migration, Max Planck Institute of Animal Behavior, Radolfzell, Germany; ^3^ Department of Biology, University of Konstanz, Konstanz, Germany; ^4^ Max Planck Institute of Animal Behavior, Radolfzell, Germany

**Keywords:** anthropogenic disturbances, anti-predatory response, flight initiation distance, urban mosaic

## Abstract

Human-induced disturbances affect animal behaviours such as anti-predatory responses. Animals in urban environments tend to exhibit a reduced escape response, measured as a shorter flight initiation distance (FID), compared to their rural counterparts. While FID has been evaluated in animals dwelling in contrasting habitats (e.g. urban versus rural), little is known about how this response varies within urban environments, especially in tropical cities. Here, we studied the FID of 15 resident bird species in Bogota, Colombia, at 22 sites grouped into four categories (natural sites, metropolitan parks, zonal parks and residential areas) that differed in landscape features and evaluated which factors affected the escape responses of birds. We showed that birds foraging in larger flocks are more tolerant when being approached but they do not seem to be influenced by other factors such as heterospecific flock size, noise levels, pedestrian density, predator density, natural cover or body length. Also, birds inhabiting residential areas and parks showed a shorter FID compared to birds in natural areas suggesting that they are more tolerant of human-related disturbances compared to their conspecifics that live in natural areas within the city. Our study shows important differences in bird anti-predatory responses within the city and suggests that social strategies (i.e. flocking patterns) may be a mechanism for adapting to human-induced disturbances in urban tropical environments.

## 1. Introduction

The behaviour and physiology of animals is affected considerably by urbanization [1–3]. For example, urban birds are usually more tolerant of environmental stimuli, such as approaching humans and anthropogenic disturbances, compared to their rural counterparts (i.e. changes in anti-predatory behaviour) [4–6]. However, while comparing contrasting habitats (i.e. urban versus rural) can reveal the effects of urbanization, few studies have considered how the landscape heterogeneity within cities [7] affects the perception of risk and escape decisions of urban birds [8,9]. In areas with different levels of vegetation cover (i.e. from urban wildlife reserves to bare concrete zones) and building density (i.e. suburbs to densely populated zones), the response of animals to human-induced disturbances could depend on the perceived risk [10]. Thus, understanding which factors affect risk perception in human-modified environments allows us to understand the mechanisms by which species cope with urbanization.

Recent studies conducted in temperate areas (i.e. Europe, Australia and the United States) suggest that birds in highly human-intervened environments exhibit a more tolerant response towards humans [4,6,9]. Tolerance towards humans has often been assessed by calculating animals' flight initiation distance (FID)—the distance at which an individual responds to a human approaching by foot [11]. FID is mainly influenced by factors related to the life-history traits of the species and the characteristics of the habitat in which they dwell. Evidence suggests that animals are more tolerant of human presence (i.e. shorter FID) when they: 1) belong to species with shorter body size [12], 2) are residents [13], 3) are less gregarious (i.e. tend to forage in smaller groups) [14,15], 4) are exposed to higher pedestrian densities [16], 5) are seldom exposed to domestic animals [17] and 6) inhabit areas with increased noise pollution [18] and with a high human-intervention level (i.e. urban streets and lawns) [9]. However, how this response varies within cities is a topic that is still understudied.

We quantified the FID of tropical urban birds exposed to different levels of human-induced disturbances, a topic understudied in the neotropics, in habitats with different levels of urbanization in Bogota, Colombia. We aimed to understand: 1) which factors influenced anti-predatory responses in urban tropical birds, and 2) whether anti-predatory responses varied across habitats within a tropical city. We hypothesized that birds would be more tolerant of approaching humans when exposed to higher environmental stimuli [4]. Thus, we expected FID to decrease: 1) as environmental noise level, pedestrian density and predator density increases and 2) from natural to residential areas. When assessing factors related to the patterns of association and body length (a proxy for body size) of birds, we expected FID to: 1) decrease as flock size increases, because birds in larger flocks have a lower individual predation risk which can result in a ‘less cautious’ behaviour [19], and 2) increase as body length increases because this pattern has been reported previously [4,12]. Lastly, since urbanization might ‘filter’ species based on their life-history and biological traits [20], we predicted, based on behavioural characteristics of the species and eBird reports (https://ebird.org) (combined with *personal* observations), that only a few species (Rock dove (*Columba livia*), Sparkling violetear (*Colibri coruscans*), Eared dove (*Zenaida auriculata*), Great thrush (*Turdus fuscater*) and Rufous-collared sparrow (*Zonotrichia capensis*)), would dwell in all of the evaluated habitats. As for the FID variation among these species, we expected it to behave according to their body length (i.e. to decrease as body length decreases).

## 2. Material and methods

### 2.1. Study area

The study was carried out in Bogota, Colombia, which is a highly urbanized area [21], with high population density [22], a heterogeneous landscape [23] and high avian diversity [24]. We categorized 22 sites based on the characterization of their vegetation cover extension (i.e. scarce, medium or high vegetation cover) and their patterns of human use, as defined by *Secretaria de Planeación Distrital* in [25]. We defined four categories: 1) *Natural sites*, areas with relatively high vegetation cover where restricted recreational activities are allowed. In our case, these areas were all wetlands. 2) *Metropolitan parks*, large areas with medium vegetation cover and recreational infrastructure, which are highly visited by the public and have restrictions to domestic animals. 3) *Zonal parks*, areas that are mainly used by nearby populations with less restrictive rules for domestic animals and 4) *Residential areas*, which have low or no vegetation cover, high infrastructure and regular pedestrian and vehicular transit (electronic supplementary material, table S1). Sites were selected to ensure a balanced sample size across categories (achieved for zonal parks, metropolitan parks and residential areas). However, in the case of natural sites, our selection was limited to areas that were open to the public (due to pandemic restrictions), easily accessible and safe to visit.

### 2.2. Data collection

#### 2.2.1. Quantifying anti-predatory responses using FID

We measured anti-predatory responses as the flight initiation distance of urban birds in 22 study sites: three natural sites, six metropolitan parks, six zonal parks and seven residential areas (electronic supplementary material, figure S1). We sampled each zonal park, metropolitan park and residential area twice and each natural site four times. Since natural sites had fewer replicates than the other categories, we sampled sites from this category more times to balance sampling effort among categories.

At each site, we quantified the FID following [26]. To do this, the observer (MAV) first walked, following random transects, around the study sites to identify the focal birds. A bird was considered focal if it had not been disturbed by the observer, other pedestrians or a predator, was foraging alone or in flocks and, was within a 30 m range (modified from [27]). The observer signalled the starting position with a marker (the distance between bird and the position where the observer first located the bird; the starting distance; SD). Next, the observer walked towards the focal individual at a constant speed (0.5–1 m s^−1^) without losing sight of the bird. When the focal bird exhibited an alert behaviour (i.e. bird extends its neck vertically and directs its attention to the observer), the observer signalled it with another marker without interrupting its path (the alert distance; AD). Once the focal bird exhibited an escape behaviour, the observer marked the place (the distance walked by the observer; DW), and then walked to the location where the focal individual was at the start of the experimental approach to calculate SD, AD and DW. We did FID trials opportunistically on all bird species that were available during the sampling session and that met the conditions for being a focal individual.

For each FID trial, the observer recorded the SD, AD, DW and the escape strategy that birds exhibited (walked fast, walked away, hopped, flew, swam away). Distances were measured using a laser rangefinder (BOSCH GLM 20) when possible or through previously calibrated paces. Finally, the FID was calculated as the starting distance (SD) minus the distance walked by the observer (DW). To estimate the FID of focal birds that were perching in shrubs or trees (i.e. birds perched up to 5 m on trees or bushes), the observer visually estimated the perch height using their own height as a reference. We calculated the horizontal FID (i.e. FID from ground foraging birds) of birds perching in trees by applying the corrected formula reported by [12] to transform vertical FIDs (i.e. FID from birds in trees) into horizontal FIDs.

Sampling sessions were carried out between 7 and 10 AM, from February to June 2021. To reduce human errors, the observer trained distance estimation, pace calibration (i.e. maintaining a consistent pace length while walking toward a focal bird) and birds' alert and escape behaviour recognition routinely for a month (January 2021) before data collection began. To train pace calibration, the observer estimated the distance between herself and a random object through paces (average of observer's pace length in training sessions: 0.6 m) and then corroborated the measure with the laser range finder. Pace calibration was achieved when the difference between the estimated distance and the measured distance was less than 0.5 m. To avoid data replication (i.e. sampling the same bird twice) the observer sampled birds were at least 25 m apart in any given sampling session.

#### 2.2.2. Factors that affect the anti-predatory response

We measured factors associated with risk-perception that are known to affect birds' anti-predatory response in human-intervened ecosystems [4,15,28]. We evaluated: 1) Body length of the focal species, a proxy for body size, extracted from [29], 2) Hetero and conspecific foraging flock size, the number of hetero-specific and conspecific birds within a 10-m radius from the focal individual [8], 3) Environmental noise level (decibels, dB), recorded using a sound level meter (UNI-T UT353-BT), 4) Pedestrian density, the number of humans that walked by the study site in a 15-min interval [16], 5) Predator density, the number of domestic animals and/or raptors that walked/flew by the study site in a 15-min interval [16] and 6) Natural cover, percentage of vegetation (i.e. trees, shrubs and lawn) and water bodies (i.e. lakes, lagoons and rivers) in each study site (see below). We measured flock size at the start of the trial and noise level and pedestrian and predator density three times during a sampling session at different places of the study site to generate a sampling day average.

#### 2.2.3. Quantification of natural cover

We conducted a supervised image classification using the software QGis v. 3.22 [30] to quantify the amount of natural cover. We used a LANDSAT 8 satellite's image of Bogota, to generate a natural colour composite raster using the bands 4, 3 and 2 [31]. We generated 30 points that were classified in three categories: 1) urban (white to grey pixels), 2) vegetation (green pixels) and 3) water bodies (dark blue or black pixels). We ran a supervised classification using the dsetzaka classification tool [32] and quantified the number of hectares using the tool r.report in QGis [33]. We obtained the total number of hectares of each cover category for each study site (i.e. urban, vegetation and natural cover) and calculated the percentage of each cover category for each study site. We defined natural cover as areas of escape, which included vegetation cover and water bodies.

### 2.3. Statistical analysis

We conducted our analyses in three steps. First, we verified that our site categories differed among them in the four measures related to the level of anthropogenic disturbance of the sites: environmental noise level, pedestrian density, predator density and natural cover percentage (see section ‘*Do site categories differ in the level of anthropogenic disturbance?*’). Second, we identified the factors that affected FID of the sampled species (see section ‘*Which factors influence anti*-*predatory response* *in urban tropical birds?*’) and third, we evaluated if FID varied among categories (see section ‘*How do anti-predatory responses vary in an urbanization mosaic?*’).

For the last two steps, we first subset the data according to the species that we were going to include in the models as explained in their corresponding sections. We also tested if there was a strong phylogenetic signal in the FID (see procedure in section ‘*Which factors influence anti-predatory response in urban tropical birds?*’) to avoid statistical dependence in the data points due to phylogenetic relatedness among species [34] and reduce the rate of Type I errors by accounting for phylogeny in the absence of a phylogenetic signal [35] in the built models. We did not find a strong phylogenetic signal in the second step (i.e. factors that affected the FID of birds); therefore, we fitted a Linear Mixed Effects Model (LMM). Contrarily, we found a strong phylogenetic signal in the third step (i.e. variation of the FID in the urbanization mosaic) and thus we fitted a Bayesian regression model (Brm). In both regression models, we added the number of observations per species as a weighted factor to control for differences in sampling effort and included study site and species as random effects. All analyses were done in R v. 4.1.2 [36].

#### 2.3.1. Do site categories differ in the level of anthropogenic disturbance?

We performed four one-way ANOVA tests, one for each of the measures of anthropogenic disturbance (pedestrian density, environmental noise level, predator density and natural cover percentage) to determine if the measures related to the level of human intervention differed among the site categories. We fitted the measure of anthropogenic disturbance as a function of the site category in each of the ANOVA tests. These analyses were performed using the aov function from the ‘stats’ package v. 4.1.2 from base R [36].

We then performed four non-planned *post hoc* Tukey multiple comparisons of means pairwise comparison, one for each of the performed ANOVAs, among site categories to determine which categories were different from each other in terms of the landscape features related to the level of anthropogenic disturbances. These analyses were also performed using the ‘stats’ package v. 4.1.2 from base R [36].

#### 2.3.2. Which factors influence anti-predatory response in urban tropical birds?

For this model, we included the species with three or more FID observations in our dataset (i.e. 15 species) since, when fitting regressions, individuals with few (one or two) observations are usually not informative and could reduce statistical power [37]. Then, we tested if there was a phylogenetic signal in the FID, using the phylosig function in the ‘phytools’ package v. 1.0–1 [38]. As phylogenetic information source, we used a maximum clade credibility tree with the maxCladeCred function from the ‘phangorn’ package v. 2.8.1 [39] (using 100 trees randomly sampled from birdtree.org using the Ericson backbone to account for the uncertainties in topology and branch length [40]). We used the Blomberg's *k*-value as a measure of the strength of the phylogenetic signal. *K*-values > 1 suggest a strong phylogenetic signal (supporting the use of models that account for the phylogenetic structure in residuals) and *k*-values < 1 suggest the absence of phylogenetic signal, justifying the use of models that do not account for the phylogenetic structure such as Linear Mixed Effects Models [41].

We did not find a strong phylogenetic signal in this model (*k*-value = 0.354), therefore, we fitted a Linear Mixed Effects Model (LMM) using the ‘lme4’ package v. 1.1–30 [42] and the ‘lmerTest’ package v. 3. 1–3 [43] to calculate *p*-values. We included log_10_-transformed FID as the response variable and untransformed starting distance, heterospecific flock size, conspecific flock size, body length, environmental noise level, pedestrian density, predator density and natural cover percentage as covariates.

We fitted the LMM with a Gaussian error structure and centred and standardized (i.e. scaled) the predictors to obtain effect size estimates that are comparable to Pearson's correlation coefficients [44]. To test for collinearity among the fitted variables, we calculated the variance inflation of the LMM with the function vif from the ‘car’ package v. 3.1–0 [45]. We removed total flock size (i.e. conspecific flock size+heterospecific flock size) from the initial model because it had a high VIF value (42.667). Lastly, we did not include the variable alert distance (AD) in the initial model because it had a strong correlation with the starting distance (SD) (*r* = 0.838, *N* = 848).

#### 2.3.3. How do anti-predatory responses vary in an urbanization mosaic?

In this model we included the species that were found in all the site categories (i.e. three species). Similar to the previous analysis, we first measured the phylogenetic signal (see previous section for the procedure). We found a strong phylogenetic signal (*k*-value = 1.28) and therefore we fitted a multi-predictor Bayesian phylogenetically informed regression model (Brm) using the ‘brms’ package v. 2.17.0 [46]. We fitted log_10_-transformed FID as the response variable and site category as the covariate. To model phylogenetic effects, we included a phylogenetic covariance matrix made with the maximum clade credibility tree from the three species constructed with the vcv.phylo function from the ‘ape’ package v. 5.6–1 [47]. We fitted a Gaussian model structure and ran four Markov Chain Monte Carlo chains with default priors (i.e. uninformative priors) (following [48]). Also, to minimize divergent transitions, we set the target average proposal acceptance probability to 0.999 and the maximum tree depth to 20 (modified from [48]). Lastly, we calculated the conditional *R*^2^ and marginal *R*^2^ to determine how much of the variance was explained by both fixed and random effects (conditional *R*^2^) and only by the fixed factors (marginal *R*^2^) using the b2_bayes function from the ‘performance’ package v. 0.9.1 [49]. Statistical significance of the Bayesian regression model was determined if the credible intervals of the analysed variable did not contain zero.

We then performed two non-planned *post hoc* pairwise comparisons. The first pairwise comparison was among site categories to determine which categories were different from each other in terms of FID (i.e. main effect of urbanization). The second comparison was among the species found in all the categories to determine if FID varied between them (i.e. main effect of species). Both comparisons were done using the ‘emmeans’ package v. 1.7.2 [50].

## 3. Results

We collected 855 FID measures on 20 bird species from 12 families and 7 orders (electronic supplementary material, table S2). The mean FID of all sampled species was 3.23 m ± 1.73, and the mean starting distance was 16.77 m ± 5.45. Our dataset for the first regression model (after removing species with one or two observations) consisted of 848 measures from 15 bird species from 11 families and 7 orders, that were distributed as follows: 205 in natural sites, 201 in metropolitan parks, 230 in zonal parks and 212 in residential areas. From the 15 species in this study only three (of the five species predicted) were found in all the evaluated site categories: Eared dove (*Zenaida auriculata*), Great thrush (*Turdus fuscater*) and Rufous-collared sparrow (*Zonotrichia capensis*). They accounted for the majority of the observations (*N* = 638).

### 3.1. Site categories differed in the level of anthropogenic disturbance

**Figure 1.**
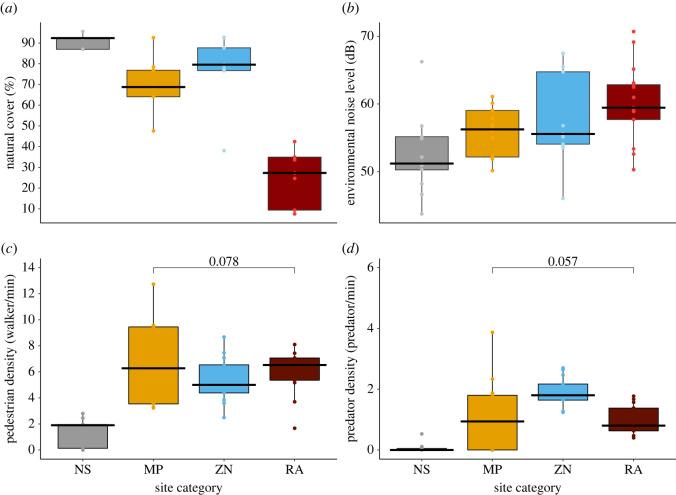
Differences in anthropogenic disturbance measures: (*a*) natural cover, (*b*) environmental noise level, (*c*) pedestrian density and (*d*) predator density between site categories. Natural sites (grey), metropolitan parks (orange), zonal parks (blue) and residential areas (dark red). In each box, the line in the middle indicates the mean value of the variable, whiskers indicate the standard deviation, and dots indicate the non-repeated values from the raw data. In each of the measures, the performed *post hoc* pairwise comparisons indicated significant differences among all pairs of site categories except from the pairs indicated with brackets. The *p*-value of these comparisons is placed above the brackets. The comparisons are reported in the electronic supplementary material, table S3.

Our site categories varied in the level of anthropogenic disturbance (figure 1, table 1, electronic supplementary material, table S3). Natural sites showed a lower pedestrian and predator density, and environmental noise level when compared to the other site categories (figure 1, electronic supplementary material, table S4). Parks were characterized by a higher pedestrian and predator density, and environmental noise level than natural sites (figure 1, electronic supplementary material, table S4). Residential areas had a higher pedestrian density and environmental noise level but a lower predator density and natural cover than parks (figure 1, electronic supplementary material, table S4). Categories also differed in the percentage of urbanized area: residential areas exhibited the largest urbanization percentage (74.29 ± 13.09%) followed by metropolitan parks (28.61 ± 15.2%), zonal parks (24.58 ± 19.33%) and natural sites (8.38 ± 4.33) (electronic supplementary material, table S4).

**Table 1.** ANOVAs results indicating differences in the variation in environmental noise level, pedestrian density, predator density and natural cover percentage, associated to the level of anthropogenic disturbance between site categories. Sum of squares, mean of squares, degrees of freedom (d.f.), F-statistic value and *p*-values of the four tests performed (one for each of the measures) are reported. Residuals are reported below each model in italics. Bold values indicate significant results at a 95% confidence level.

measure	d.f.	sum of squares	mean of squares	*F* value	*p*-value
environmental noise level	3	1 230 794	410 265	69.99	**<2 × 10^−16^**
*residuals*	*844*	*4* *988* *846*	*5911*		
pedestrian density	3	710 985	236 995	295.9	**<2 × 10^−16^**
*residuals*	*844*	*673* *285*	*798*		
predator density	3	77 768	25 923	249.5	**<2 × 10^−16^**
*residuals*	*844*	*86* *732*	*103*		
natural cover percentage	3	103 264 843	34 421 614	994.8	**<2 × 10^−16^**
*residuals*	*844*	*28* *977* *196*	*34* *333*		

### 3.2. Birds that foraged in larger conspecific flocks had shorter FIDs

**Figure 2.**
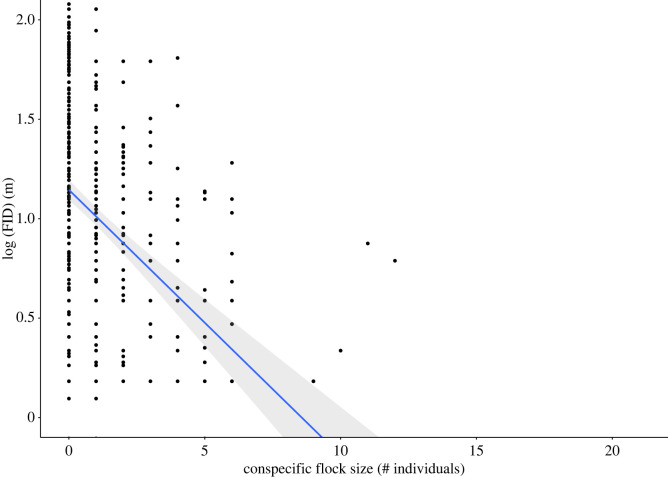
Relationship between conspecific foraging flock size and log_10_-trasnformed flight initiation distance (*N* = 848). The plot shows the regression line for the main effect (blue line) and its corresponding 95% confidence interval (shaded zone). Dots represent the raw data log-transformed, and values in the axis labels have been back-transformed for readability.

We found a significant negative correlation between FID and conspecific foraging flock size (table 2). Birds that were foraging in larger conspecific flocks showed a more tolerant response when approached by humans (i.e. shorter FID) compared to birds foraging in smaller flocks or by themselves (figure 2). FID showed a tendency to decrease as pedestrian density, body length, heterospecific flock size and noise increased and a tendency to increase as predator density and natural cover increased. Note that none of these relationships were statistically significant (table 2).

**Table 2.** LMM coefficients and standard errors from linear-mixed models evaluating the influence of environmental noise level, pedestrian density, predator density, body length, conspecific flock size, heterospecific flock size and natural cover percentage (fixed effects) on flight initiation distance (FID). Bold values indicate statistical significance at *α* = 0.05. *N* = 848 corresponds to the total number of FIDs included in the regression model. The variance explained by the model was *R*^2^ = 0.002.

fixed effect	estimate	S.E.	*t*-value	*p-value*
**intercept**	−0.091	0.195	−0.47	0.648
starting distance	**0.106**	**0.026**	**4.104**	**4.45** **× 10^−5^**
**heterospecific flock size**	−0.026	0.026	−1.021	0.31
conspecific flock size	**−0.285**	**0.026**	**−11.068**	**<2** **× 10^−16^**
**body length**	−0.14	0.144	- −0.976	0.34
**environmental noise level**	−0.078	0.04	- −1.943	0.052
**pedestrian density**	0.067	0.054	1.231	0.219
**predator density**	0.002	0.07	0.024	0.981
**natural cover percentage**	0.182	0.09	2.031	0.055
***random effect***	variance	S.E.		
**study site**	0.158	0.398		
**species**	0.153	0.392		
**residual**	96.688	9.833

### 3.3. Birds in natural sites exhibited a greater FID compared with birds in residential areas and parks

**Figure 3.**
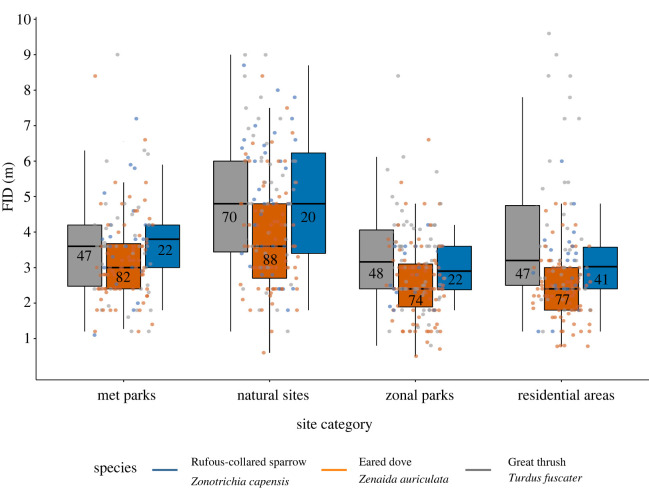
Urbanization influence on flight initiation distance (FID). Colour dots represent FID raw data (*N* = 638) of each of the species that were in all the site categories: eared-dove in orange, rufous-collared sparrow in blue and great thrush in grey. The plot shows the medians (black line inside the box) with boxes showing the lower and upper quartile and black vertical bars showing the full range of the data. Numbers in blue inside of the box indicate the number of FID trials per site category (*N* = 638: natural sites = 165, metropolitan parks = 144, zonal parks = 178, residential areas = 151).

We found that FID in natural sites differed from all other evaluated site categories (figure 3; table 3; electronic supplementary material, table S5). Birds found in natural sites exhibited longer escape responses than birds in zonal parks, metropolitan parks or residential areas. This suggests that landscape features affect birds' risk perception and therefore, its anti-predatory responses. From the three species found in every site category, the FID of the eared dove (the medium-sized species) differed from the escape responses of the other two species (electronic supplementary material, figure S2). FID did not vary significantly between the smallest species (the rufous-collared sparrow) and the largest species (the great thrush) (electronic supplementary material, figure S2). As a general trend, the eared dove exhibited the shortest escape response across all sites and the great thrush (the largest species) exhibited the longest escape response (electronic supplementary material, figure S2).

**Table 3.** Brm coefficients and standard errors from multi-predictor Bayesian phylogenetically informed regression model evaluating the influence of urbanization mosaic on the flight initiation distance (FID) of the three species that were represented in every site category. The reference level for the site categories was metropolitan park. The total number of FIDs included in the model N_T. fuscater_ = 212; N_Z. capensis_ = 105; N_Z. auriculata_ = 321 (in total *N* = 638). Bold values indicate significant results (i.e. credible intervals that did not contain zero). Conditional (i.e. variance explained by both fixed and random effects) and marginal (i.e. variance explained only by fixed effects) R^2^ are also reported here. The posterior phylogenetic signal of the brm was *λ* = 0.03.

fixed effect	estimate	S.E.	lower 95% CI	upper 95% CI
intercept	**1.19**	**0.43**	**0.43**	**1.99**
natural sites	**0.37**	**0.15**	**0.08**	**0.66**
residential areas	**−0.23**	**0.12**	**−0.46**	**−0.00**
zonal parks	−0.21	0.12	−0.44	0.03
*random effect*				
species	0.05	0.08	0.01	0.25
study site	0.21	0.04	0.15	0.30
conditional r2	0.334		0.331	0.337
marginal r2	0.231		0.102	0.357

## 4. Discussion

We showed that the escape behaviour of urban tropical birds is linked to the variation in conspecific flock size. FID decreased when individuals foraged in larger conspecific flocks, suggesting a flight-delayed strategy. Additionally, the relationship between FID and urbanization levels analysed with a model that considered phylogenetic relationships showed that birds that inhabit areas with less human intervention within cities, such as wetland reserves, were less tolerant when being approached compared to birds that used other types of habitats (i.e. parks and residential areas). Our study suggests that social strategies such as the formation of conspecific groups may facilitate the adaptation of individuals to life in urban ecosystems and highlights the importance of natural areas (i.e. protected reserves) within the city.

The decrease of FID as group size increases supports the idea that sociality is an important predictor of predation risk and thus escape decisions [51]. Birds in larger flocks experience a reduced probability of predation [52]. The observed response indicated that birds may form large flocks as a strategy to reduce the risk of predation which could be the result of a dilution effect (i.e. the ‘predation dilution’ hypothesis; [19]) and may indicate a flight-delaying strategy that allow birds to maximize the benefits of delayed escape such as an increase in food intake [53]. However, the relationship between FID and group size varies across taxa [28] and other studies in temperate areas have shown that birds in larger groups exhibit an increase in FID when being approached by humans [14,15,54] (i.e. the ‘many eyes’ hypothesis; [55]). This suggests that there might be important differences in the mechanisms to cope with human-induced disturbances between tropical and temperate species, potentially as a result from differences in life-history traits, exposure to predation and past experiences with humans.

Despite our considerable dataset, we did not find a relationship between FID and body length, pedestrian density, predator density, environmental noise level or natural cover. The lack of an association between FID and body length was especially surprising given that: 1) body length, a proxy for body size, has been reported as an important variable influencing variation in FID [4,12], 2) our data presented a wide range of body lengths (range = 10.2–75 cm), although there was not an even representation for all body lengths (electronic supplementary material, table S2), and 3) this relationship was reported in a previous study conducted in a neotropical area in Brazil [56]. Larger species may experience higher risk of predation compared to smaller ones (i.e. species that exhibit shorter FIDs), which indicates variation in the cost of staying among birds with different body length [12]. However, our data suggest that under intense human-induced disturbances these differences may disappear, a hypothesis that needs to be further explored.

Urbanization has been identified as an important factor that affects FID in birds [4,6,57]. Our study showed that birds in residential areas and parks were less responsive (i.e. shorter FID) than birds in urban wetland reserves (i.e. natural sites), which suggests that the extension of natural vegetation cover might be an important factor in the escape decisions of urban tropical birds. These results were in line with previous studies [8,9] including the study conducted in a neotropical area in Brazil [56], showing that birds in natural vegetation had longer FIDs than birds in more urbanized environments. Moreover, a reduced fear response can result from habituation, plasticity or adaptation to anthropogenic disturbances, and can facilitate the colonization of urban environments [9]. However, a greater response toward human approaches (i.e. longer FID) could also result from other life-history traits that we did not consider or the personality of the focal individual. Personality can affect FID in several ways [58–61], thus it is possible that within cities, birds that inhabit less human-intervened sites are less bold than the ones that live in fully urban zones and exhibited larger escape responses [62]. Moreover, birds in parks exhibited a similar response to the birds found in residential areas. This might be because, although parks had more vegetation cover than residential areas, they still had a high pedestrian density, predator density and environmental noise level (figure 1). Therefore, birds in these areas are also exposed to high frequency and intensity of stimuli which can result in similar escape responses due to acclimation to anthropogenic disturbances [11,63].

Variation between species has been reported by previous studies [16,64]. In our study, escape responses varied between the eared dove and the great thrush and the rufous-collared sparrow. However, this variation did not follow our prediction since the medium species exhibited the shortest escape response (i.e. we expected FID to decrease as body length decreases; thus, we expected the smallest species to exhibit the shortest FID). These differences can result from variation in life-history traits that we did not consider in our study and are expected to affect animals' responses to human approaches [12], and thus, how species respond to anthropogenic disturbances. We found that the eared dove showed the shortest FID across all study sites. The eared dove is considered a neophyllic species [65], which could explain why it is a dominant species in several neotropical cities [66,67] and its success in human-modified environments [66]. Also, human attitudes towards birds could also affect escape decisions [8,68]. For instance, humans feed doves more often in residential areas than in other habitats (MAV personal observation). This human–wildlife interaction may increase their tolerance of human presence [68] and reduce their FID, although the mechanisms and consequences of such human–wildlife interaction are yet to be studied. Lastly, phylogenetic relatedness between the great thrush and the rufous-collared sparrow could result in a similar response to human approaches. Alternatively, these species may be neophobic and therefore are timid when being approached. However, we did not find studies supporting these hypotheses, and thus they need further exploration.

Anti-predatory behaviour of urban wildlife has been mainly studied in temperate areas, thus little is known about this behaviour and its drivers in tropical wildlife. Tropical and temperate species differ in behaviour, ecology and life-history traits [69] that may influence their risk perception. Thus, their anti-predatory response was expected to differ. Although we did not explicitly test for differences between tropical and temperate birds, we expected tropical birds to exhibit longer escape distances than temperate ones because, compared to temperate birds, tropical birds have a greater future reproductive potential and a slow pace-of-life [70]. However, our results suggested differently. For example, within the genus (*Turdus*), the tropical great thrush exhibited a shorter FID (mean_FID_ = 3.54 m ± 1.63) compared to the temperate common blackbird (mean_FID_ = 4.92 m ± 3.35) [16], suggesting a greater effect of urbanization on escape responses. The rock dove, the only species in our dataset that can be found in both temperate and tropical habitats, exhibited a shorter escape distance (mean_FID_ = 1.64 m ± 0.75) in Bogota, compared to individuals from temperate zone cities (mean_FID_ = 5.3 m ± 0.4; [71], 2.78 m ± 1.61; [16]). This suggests that the differences in FID are rather due to the frequency and intensity of human-induced disturbances (stimuli), which can be higher in tropical cities because of poor urban planning [22]. As a result, birds found in highly disturbed tropical habitats might be more tolerant of approaching pedestrians [5,8,9]. Nonetheless, it is important to note that the available data to support this observation is still scarce. Further research is necessary to understand the differences between tropical and temperate birds and the extent to which habitat characteristics play a more important role than life-history traits in regulating escape responses to human-induced disturbances.

## 5. Conclusion

Our study makes a considerable contribution to the understanding of the variation and drivers of anti-predatory responses in tropical birds within urban ecosystems. Our results indicate that birds found in fully urban environments may have adapted or acclimated to human-induced disturbances (e.g. high environmental noise, pedestrian and predator density). However, acclimation can negatively affect the detection of natural predation cues (i.e. predation cues from cats or dogs) or other threats such as cars and public transportation [72]. Birds that live in natural areas (i.e. urban wildlife reserves) are less tolerant of human-induced disturbances and could be overly affected by increased recreational activities, thus highlighting the importance of natural reserves within the cities to maintain behavioural variation. Finally, although our study does not explicitly compare the response of temperate and tropical birds, it suggests that tropical birds may be under different ecological pressures than temperate birds and calls for further studies aiming to understand the differences in the escape response between species in these areas.

## Data Availability

The data and code to replicate the results can be found at https://osf.io/njzc4/. The data are provided in electronic supplementary material [73].
